# Identification, Characterization and Down-Regulation of *Cysteine Protease* Genes in Tobacco for Use in Recombinant Protein Production

**DOI:** 10.1371/journal.pone.0130556

**Published:** 2015-07-06

**Authors:** Kishor Duwadi, Ling Chen, Rima Menassa, Sangeeta Dhaubhadel

**Affiliations:** 1 Department of Biology, University of Western Ontario, London, ON, Canada; 2 Agriculture and Agri-Food Canada, 1391 Sandford Street, London, ON, Canada; Universidade Federal do Rio Grande do Sul, BRAZIL

## Abstract

Plants are an attractive host system for pharmaceutical protein production. Many therapeutic proteins have been produced and scaled up in plants at a low cost compared to the conventional microbial and animal-based systems. The main technical challenge during this process is to produce sufficient levels of recombinant proteins in plants. Low yield is generally caused by proteolytic degradation during expression and downstream processing of recombinant proteins. The yield of human therapeutic interleukin (IL)-10 produced in transgenic tobacco leaves was found to be below the critical level, and may be due to degradation by tobacco proteases. Here, we identified a total of 60 putative cysteine protease genes (*CysP*) in tobacco. Based on their predicted expression in leaf tissue, 10 candidate *CysP*s (*CysP1*-*CysP10*) were selected for further characterization. The effect of *CysP* gene silencing on IL-10 accumulation was examined in tobacco. It was found that the recombinant protein yield in tobacco could be increased by silencing *CysP6*. Transient expression of *CysP6* silencing construct also showed an increase in IL-10 accumulation in comparison to the control. Moreover, CysP6 localizes to the endoplasmic reticulum (ER), suggesting that ER may be the site of IL-10 degradation. Overall results suggest that *CysP6* is important in determining the yield of recombinant IL-10 in tobacco leaves.

## Introduction

Plants are attractive biofactories for recombinant protein production. They offer several advantages such as reduction in cost of production and increased scalability over microbial and mammalian cell culture systems. This low cost of large scale protein production is due to significant reduction in capital investment and processing cost. Unlike cell culture based systems, plants do not require fermenters or highly skilled personnel to operate them [1]. Plants are also capable of carrying out complex post translational modifications (PTM), a process necessary for the biological activity of many eukaryotic proteins. Additionally, plant systems include reduced risk of contamination from animal-borne pathogens, and provide a platform to manufacture valuable pharmaceutical proteins that cannot be produced using fermenter-based systems [2,3]

Over the past two decades, a wide range of food and non-food crops such as tobacco, alfalfa, cereals (rice and maize), legumes (soybean), and fruits and vegetables (carrot, tomato, potato) have been used for recombinant protein production [1]. Being a non-food crop, tobacco (*Nicotiana tabacum*) offers a competitive edge over many other plant hosts in terms of biosafety. Tobacco provides a high yield of protein per green leaf biomass that can be harvested 3–4 times in a year, and has a prolific seed production ability [4]. Furthermore, molecular and genetic tools for transfer and expression of foreign genes in tobacco are well established. The first pharmaceutical protein produced *in planta*, human growth hormone (hGH), was expressed in the tobacco leaves [5]. Since then, many recombinant proteins such as monoclonal antibodies, recombinant subunit vaccines and cytokines have been successfully produced in tobacco [3,6–8].

Though plant bioreactors have emerged as an economical alternative, low accumulation of proteins is the major concern posing limitations for its use as a commercial protein production platform [9]. One of the major reasons for low yields of recombinant proteins is their degradation by plant proteases [10,11]. Protein degradation by plant proteases occurs both intracellularly and extracellularly. Inside the cell, degradations can occur after synthesis, assembly and in some cases during PTM in the endoplasmic reticulum (ER) and golgi apparatus [12]. Outside the cell, proteins are degraded by extracellular proteases in the apoplastic space or in the culture medium when they are synthesized as secreted proteins [13]. Degraded protein fragments have been observed in many plant hosts expressing recombinant proteins, such as tobacco [14–17], *Arabidopsis* [14], corn [18,19], potato [20] and alfalfa [21]. Among several different classes of plant proteases, cysteine proteases (CysPs) were identified as the enzymes responsible for the degradation of recombinant sea anemone protein equistatin when expressed in potato tubers [20], and for the degradation of the human cytokine, hGM-CSF, produced in rice cell suspension cultures [22].

CysPs are ubiquitous proteins found in organisms ranging from bacteria, fungi, viruses to plants and animals. In plants, CysPs play key roles in environmental stress response, nutrient remobilization and cellular housekeeping, and account for nearly 30% of the proteolytic activity in mature organs [23]. In *Arabidopsis*, CysPs are known to carry out multifarious roles in different tissues during development [24–26]. However, not much is known regarding the role of CysPs in tobacco. Few tobacco CysPs have been characterized which are implicated in stress response [27], protein degradation during programmed cell death [28], pollen grain development [29], and amino acid remobilization in senescing leaves [23].

Interleukin (IL)-10 is an immune-regulatory cytokine produced by immune cells that may turn into a new therapeutic target for the treatment of chronic inflammatory diseases [30]. IL-10 has been used as a model recombinant protein to study the factors that control the synthesis and accumulation of recombinant proteins in plants [31]. Despite several efforts towards increasing the production of IL-10 in tobacco [32–34], its accumulation during initial stable expression in whole plants was reported to be less than 1% of the total soluble protein, a level which is not sufficient for the viable commercial production of proteins. Here using a functional genomics approach, we identified a cysteine protease, CysP6, which affects IL-10 accumulation level in tobacco leaves. Furthermore, CysP6 is localized to the ER, indicating that the ER is the potential site of IL-10 proteolysis.

## Materials and Methods

### Plant materials and growth conditions

Seeds of tobacco (*N*. *tabacum*) cv. 81V9 and the transgenic line G7 homozygous for the IL-10 transgene (hereby called as IL-10 control) were grown in a green house at 24°C and 16 h daylight, with 60–70% relative humidity. The IL-10 control tobacco line overexpresses IL-10 that is designed to accumulate in the ER [32]. *N*. *benthamiana* plants were grown in a growth chamber under similar conditions.

### *In silico* analysis

To identify *CysP* genes present in tobacco, a keyword search using “cysteine protease” was used against the DFCI tobacco gene index database (http://compbio.dfci.harvard.edu/tgi/). The database contains complete or partial *CysP* sequences obtained from sequencing of tobacco cDNA libraries. Candidate genes were selected for the study according to their expression in the leaves and their complete sequence availability in the database. The amino acid sequences of the full length *CysPs* were predicted using ExPASy software (http://web.expasy.org/translate/) and were aligned using ClustalW [35]. The domain organizations of putative CysPs were predicted using Pfam 27.0 (http://pfam.xfam.org/) and the subcellular localizations were predicted using PSORT (http://psort.hgc.jp/form.html).

### Plasmid construction

To produce RNAi expression clones targeting candidate *CysP* genes, the gene fragments were PCR amplified using gene-specific primers (S1 Table) and cloned into pDONR/Zeo vector using the BP clonase reaction mix (Invitrogen, USA), sequence confirmed, and recombined into the RNAi destination vectors pB7GWIWG2(II),0 or pK7GWIWG2D(II),0 using the LR clonase reaction mix (Invitrogen, USA). The orientation of inserted *CysP* fragments in the destination vectors was confirmed using the gene-specific forward primers (CysPF) and the vector-specific chloramphenicol resistance (*Cmr*) primers (S1 Table). The recombinant destination vectors were transformed into *A*. *tumefaciens* GV3101 and used for plant transformations.

To study the subcellular localization, full-length *CysP6* was amplified using CysP6-OE-F/R1 primers (S1 Table). The PCR product was recombined into the Gateway entry vector pDONR/Zeo, followed by recombination into the destination vector pEarlyGate101 to create a pEG101-*CysP6-YFP*.

### Tobacco transformation

For stable and transient expression of *CysP* silencing constructs, *A*. *tumefaciens* strain GV3101 harbouring the constructs were grown in infiltration culture media (LB medium with 10 mM morpholino-ethanesulfonic acid [MES] pH 5.6, 100 μM acetosyringone), supplemented with rifampicin (10 μg/mL), gentamycin (50 μg/mL), and kanamycin (50 μg/mL for pK7GWIWG2D(II),0-CysP plasmids) or spectinomycin (50 μg/mL for pB7GWIWG2(II),0-CysP plasmids). The cultures were grown to an optical density (OD600) of 0.5–0.8 and centrifuged at 3000 *g* for 30 min. The pellets were resuspended in Gamborg’s solution (3.2 g/L Gamborg’s B5 and vitamins, 20 g/L sucrose, 10 mM MES pH, 5.6, 200 μM acetosyringone) to a final OD600 of 1, and incubated at room temperature for 2 h with gentle agitation to activate the virulence gene required for transformation.

Stable transformation of tobacco leaves was performed using a leaf disc transformation method. Briefly, approximately 5 mm^2^ tobacco leaf explants were submerged in the recombinant *A*. *tumefaciens* suspension cultures, blotted on a filter paper and placed on plates containing MS medium supplemented with naphthalene acetic acid (1 mg/L) and benzyl aminopurine (98 μg/L) (MST-agar). After 2–3 days of co-culture, the leaf explants were transferred to a new MST-agar plate containing timentin (500 mg/L) and BASTA (4 mg/L). The explants were then transferred to a new MST/BASTA plate after 3 days and sub-cultured in a new plate every week until calli were produced from the transformed plant cells. After the calli differentiated into independent shoots, the shoots were cut and transferred to a rooting MST-agar medium. *CysP* silenced shoots were transferred to soil after sufficient roots were produced. Individual lines were grown in different pots and transferred to the greenhouse (T_0_). T_1_ plants were also generated for a selected number of T_0_ lines. Both T_0_ and T_1_ plants were grown together with IL-10 control plants and used for the evaluation of IL-10 levels.

For transient silencing of *CysP6*, *A*. *tumefaciences* GV3101 harbouring the silencing construct was infiltrated into 7-week old tobacco plants. Fully expanded 4^th^, 5^th^ and 6^th^ leaves from the top were selected for infiltration, and the tissues were collected four days post-infiltration for total RNA and protein extraction.

### RNA isolation, RT-PCR and qPCR

Total RNA was extracted from tobacco leaves using an RNeasy Plant Mini kit (Qiagen Inc., USA). An on-column DNase1 digestion was performed to eliminate genomic DNA contamination (Promega, USA). Total RNA (1 μg) was used for cDNA synthesis using a QuantiTect Reverse transcription Kit (Qiagen Inc., USA). A quantitative RT-PCR (qRT-PCR) was performed to check the expression of *CysP6* using SsoFast EvaGreen Supermix (Bio-Rad Laboratories, Inc., USA) and a CFX96 real-time PCR detection system (Bio-Rad Laboratories, Inc., USA). *CysP6* was amplified using the primers, 5’- CACCATATCCCTACTTCTCCTCCTC- 3’ and, 5’- CCATGTTCGACTAGCCATGACTC-3’. The amplicon was cloned into pGEM-T Easy vector (Promega, USA) and sequence verified. Tobacco *actin* (Gene bank ID: AB158612.1) was used as a reference gene and was amplified using the primers 5’-GGTTGGTATGGGTCAAAAGGATCA-3’ and 5’-GGAGCAACACGCAACTCATTG-3’.

RT-PCR was performed to check the expression of *CysP6* in tobacco after transient expression of *CysP6* silencing construct. Gene specific primers (S1 Table) were used for 36 cycles of PCR and the amplified product was visualized on a 1% agarose gel.

### Protein extraction and ELISA

Protein extraction was performed in 1X phosphate buffer saline pH 7.4 containing 0.1% Tween-20, 1mM EDTA, 100 mM sodium ascorbate, 2% PVPP, 1 mM PMSF and 100 μg leupeptin. Total soluble protein in each sample was measured using the Bradford dye reagent (Bio-Rad Laboratories Inc., USA). IL-10 levels were checked using anti-human IL-10 monoclonal antibodies in a double sandwich ELISA following manufacturer’s instructions (BD Biosciences, Canada). Purified human IL-10 was used as standards in the ELISA (BD Biosciences, Canada).

### Subcellular localization

For subcellular localization, 4–6 week-old *N*. *benthamiana* leaves were infiltrated with the *Agrobacterium* culture containing pEG101-CysP6-YFP construct. Transient expression of CysP6-YFP was visualized using a Leica TCS SP2 inverted microscope. A cyan fluorescent protein (CFP) targeted to retain the ER was used as a control to verify the probable localization of CysP6-YFP in the ER. The control construct was obtained from Arabidopsis Biological Resource Center (ABRC, clone name: ER-CK) [36]. For co-localization, *Agrobacterium* cultures containing pEG101-CysP6-YFP and pEG101-ERCFP constructs were mixed in a ratio of 1:1 and co-infiltrated into tobacco leaves followed by confocal microscopy. A 63X water immersion lens was used at an excitation wavelength of 514 nm for YFP and 434 nm for CFP. The emission wavelengths for YFP and CFP were between 530–560 nm and 460–490 nm, respectively.

## Results

### Tobacco genome contains at least 60 putative *CysPs*

An *in silico* analysis was performed using the DFCI tobacco gene index (TGI) database that contains 324,058 tobacco expressed sequence tags (ESTs). A total of 55 putative *CysP* genes were identified in the database using the key word ‘cysteine protease’. Out of 55 putative *CysP* sequences, 32 were tentative contig sequences which were created by assembling ESTs into virtual transcripts. The remaining 23 were singleton sequences, each of which represented a unique EST in the database. These putative *CysPs* were derived from tobacco cDNA libraries constructed from a variety of tissues including leaf, flower, root, whole seedling or cultured tobacco cell suspension cv Bright Yellow-2. Table 1 provides a list of putative *CysP* genes as represented by their tentative contig or singleton sequence identification numbers, their tissue specific expression and predicted protein sizes for those where complete coding regions were available. Of the 55 putative *CysP*s, 10 were selected as candidates for further study based on their complete sequence availability and expression in leaf tissue. Leaf specific CysPs were of main interest, as the principal objective of this research was to identify potential CysPs involved in determining IL-10 yield in the leaf tissue. The candidate *CysPs* were named as *CysP1* to *CysP10*.

**Table 1:** List of putative *CysPs*, tissue expression and predicted protein size of candidate CysPs selected for silencing in tobacco.

TC sequence	Most expressed in	Candidate *CysP*	Predicted Protein size (AA)	Singleton Sequence	Most expressed in	Protein size (AA)
TC122594	R,L	***CysP1 (NTCP23)***	360	BP132783	BY-2	_
TC123875	R		_	BP529990	BY-2	_
TC124565	L,F,R	***CysP2***	240	CN498801	L	_
TC124614	R,L		_	DV159633	Seed	_
TC124631	L,F,R		_	DW001815	L,R	_
TC126531	L,F,R	***CysP3***	_	AM837057	L	_
TC128311	L,F,R		_	AM829003	L	_
TC129167	L,F,R		_	AM831937	L	_
TC129618	L,F,R, BY-2	***CysP5***	360	AM791536	L	_
TC130400	R		_	AM787277	L	_
TC130996	L,F,R		_	AM846161	W	_
TC132948	L,F,R	***CysP6 (NTCP6)***	466	AM834885	W	_
TC133645	L,F,R	***CysP4***	349	AM836936	W	_
TC133786	BY-2		_	AM801664	L	_
TC135985	L,R		_	AM787303	W	_
TC139681	L,F,R		_	AM823454	W	_
TC140530	L,R,W		_	AM801795	W	_
TC141516	L,F,R	***CysP7***	355	AM844688	W	_
TC143494	L,F,R	***CysP8***	434	FG634183	L	_
TC143787	L,F,R	***CysP9***	301	FG644916	L	_
TC144748	L, R,W		_	FG167001	R	_
TC145665	L,R		_	FG172560	R	_
TC151953	W		_	FG190220	L,F,R	_
TC152484	L,F,R,W		_			
TC154564	L,R		_			
TC155175	L,F,R,W		_			
TC162422	L		_			
TC163159	L,W		_			
TC164523	L		_			
TC164665	L,F,R		_			
TC166013	Proembryo		_			
TC166795	L,F,R	***CysP10***	361			

TC, tentative contig sequence; AA, amino acid; L, leaf; F, flower; R, root; W, whole plant, BY-2, *Nicotiana tabacum cv*. Bright Yellow cell line;-, partial *CysP* sequences

In addition to the 55 putative *CysPs* identified through a database search, five other *CysP* sequences were found through a literature search [29,37,38]. Their accession numbers and the paralog tentative contig sequences are shown in Table 2. Among them, *NtCP1* was expressed only in senescing leaves while *NtCP2*, *CyP7* and *CyP8* in mature leaves. *NtCP56* was strongly expressed in anthers. The untranslated region of NtCP56 and CysP10 are not conserved suggesting these two genes are not alleles. A very high degree of amino acid identity was found between NtCP56 and CysP10 (99.17%), which indicates that they might share functional similarity or belong to the members of same gene family in tobacco.

**Table 2:** Comparison of published tobacco *CysP* sequences with tentative contig sequences from tobacco EST database.

*CysP* genes	Accession #	Closest tentative contig^a^	Amino Acid identity (%)	Reference
*NtCP1*	AY881011	TC133645 (*CysP4*)	94.84	[37]
*NtCP2*	AY881010.1	TC124565 (*CysP2*)	96.6	[37]
*CyP7*	Z13959.1	TC131927	97.8	[38]
*CyP8*	Z13964.1	TC131927	97.5	[38]
*NtCP56*	EU429306.1	TC166795 (*CysP10*)	99.17	[29]

^a^Closed tentative contig in bold letters (candidate CysPs in parenthesis) share a high degree of amino acid identity with the respective published CysP sequences

### Catalytic triad and non-contiguous ERFNIN motif are conserved in candidate CysPs

**Fig 1:**
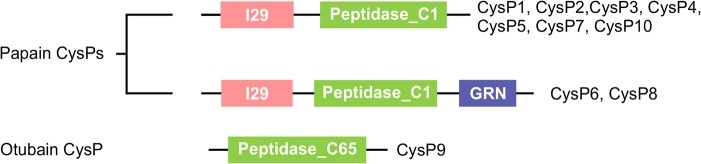
Schematic representation of different domains in papain and otubain family CysPs. Candidate CysPs having specific domain types are indicated. The diagram is not drawn to scale. I29, Cathepsin propeptide inhibitor domain; Peptidase_C1, Papain family domain; GRN, Granulin like repeats; Peptidase_C65, Otubain family domain.

To determine the domain composition of the candidate CysP proteins in [32] tobacco, we performed the domain analysis using pfam database (http://pfam.xfam.org/). The analysis revealed that CysP1-CysP8 and CysP10 each contain a common papain domain, shown to be responsible for the endo-peptidase activity of CysPs. Proteins containing such domains are classified under the papain or peptidase_C1 family and are synthesized as precursor CysPs [39,40]. Precursor CysPs have an additional propeptide sequence preceding their papain domain, called I29 or the cathepsin propeptide inhibitor domain (Fig 1). CysP6 and CysP8 have an additional granulin-like repeat that is known to play a role in targeting and activity regulation of certain CysPs [41]. CysP9 contains a single large ovarian tumor (OTU) domain suggesting it may belong to the otubain or peptidase_C65, and possibly possess functions similar to deubiquitinating enzymes of the peptidase_C65 family. Detailed information regarding putative domain structure of all candidate CysPs is shown in Table 3.

**Table 3:** Detail domain information of candidate tobacco CysPs.

Candidate CysPs	Cathepsin propeptide inhibitor domain	Papain family CysP domain	Granulin like repeats	Otubain domain
CysP1(NTCP23)	61–117	143–358	X	X
CysP2	Cleaved	5–221	X	X
CysP3	53–110	141–246*	X	X
CysP4	42–99	131–348	X	X
CysP5	61–117	143–358	X	X
CysP6(NTCP6)	50–107	138–353	387–435	X
CysP7	49–105	137–352	X	X
CysP8	1–41	75–292	339–387	X
CysP9	X	X	X	45–300
CysP10	38–93	126–342	X	X

Numbers in the domains represent amino acids.

*complete coding region sequence was not available.

X, absent in respective CysPs.

**Fig 2:**
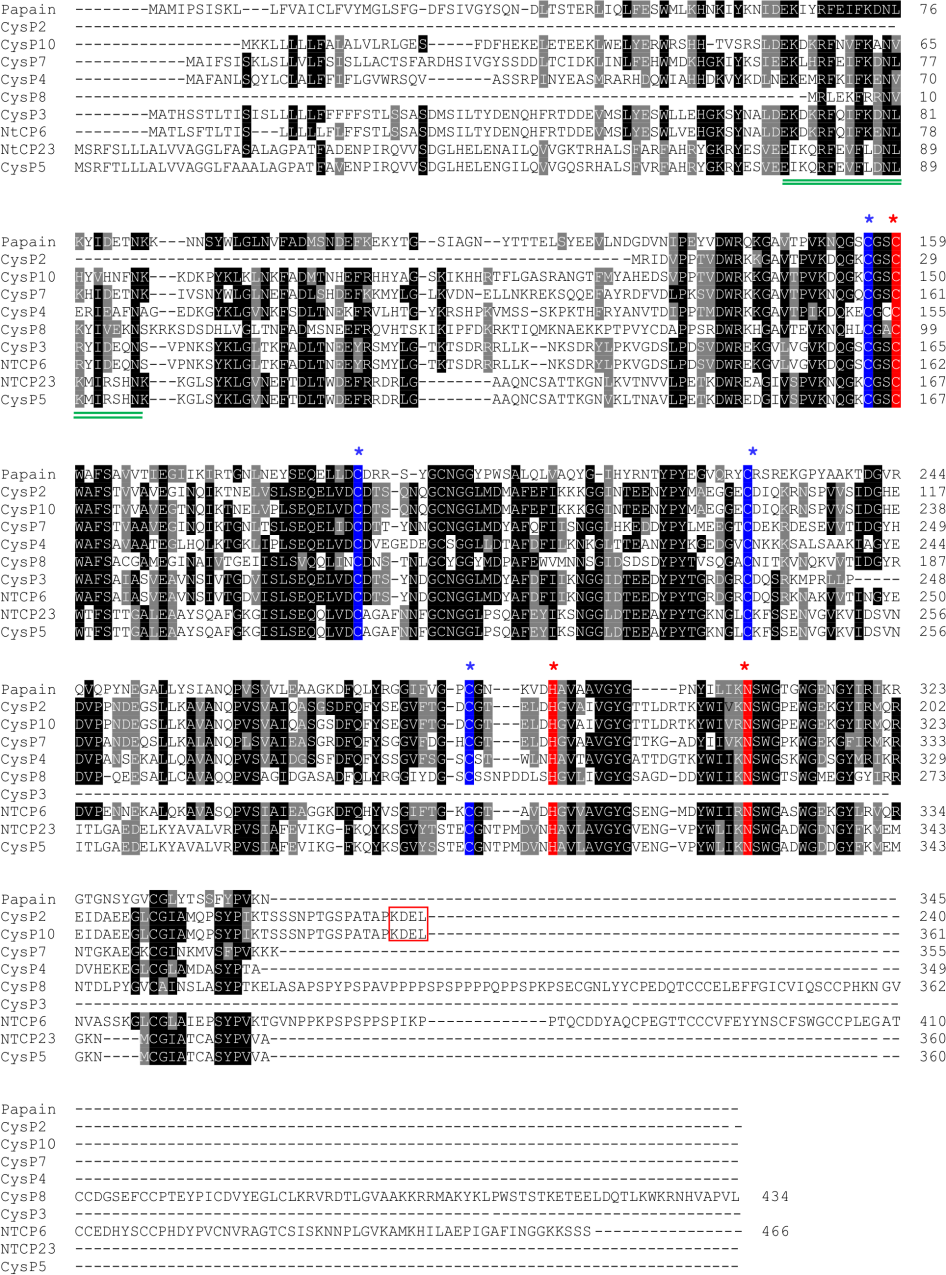
Multiple sequence alignment of Peptidase_C1 (Papain) family candidate CysPs. Predicted amino acid sequences of a papain, NTCP23 (CysP1), CysP2, CysP4, CysP5, NTCP6 (CysP6), CysP7, CysP8 and CysP10 were aligned using CLUSTALW and shaded using BOXSHADE 3.21. Identical and conserved amino acids are shaded by dark and light grey, respectively. (*) indicates catalytic residues cysteine (C), histidine (H) and asparagine (N). (*) indicates the disulfide bridge forming cysteine residues. Red box indicates endoplasmic reticulum retention signal KDEL in CysP2 and CysP10. The conserved ERFNIN motif in the propeptide region is double-underlined. C-terminal amino acids of CysP6 and CysP8 beyond the positions 410 and 362, respectively, are not shown in the alignment.

To identify catalytic residues in the CysPs, the deduced amino acid sequences of papain family CysPs identified in this study were aligned, along with papain and a previously characterized tobacco CysP, NTCP23 (Fig 2). A catalytic triad comprised of Cys25, His159 and Asn175 (papain numbering) was found in the papain domain of all aligned CysPs. Enzymatic activity of a CysP is dependent on Cys25 and His159 residues, which exist as ion-pairs in a pH interval of 3.5–8 [42]. Besides the catalytic Cys residues, four other Cys residues were also identified to be conserved in the papain domain of all aligned CysPs. These residues are likely involved in disulfide bridge formation leading to proper folding and activity of the enzymes. The alignment shown in Fig 2 excludes CysP3 as it lacked several N-terminal amino acid residues, and critical residues Asn and His in the catalytic triad. A highly conserved block of amino acids interspersed with variable residues, **E**X_3_ **R**X_3_ **F**X_2_ **N**X_3_ **I/V**X_3_ **N (**ERFNIN motif) is present in the propeptide region of all papain family CysPs [43].

### RNAi silencing of candidate *CysP* genes and generation of stable *CysP* silenced tobacco lines

**Fig 3:**
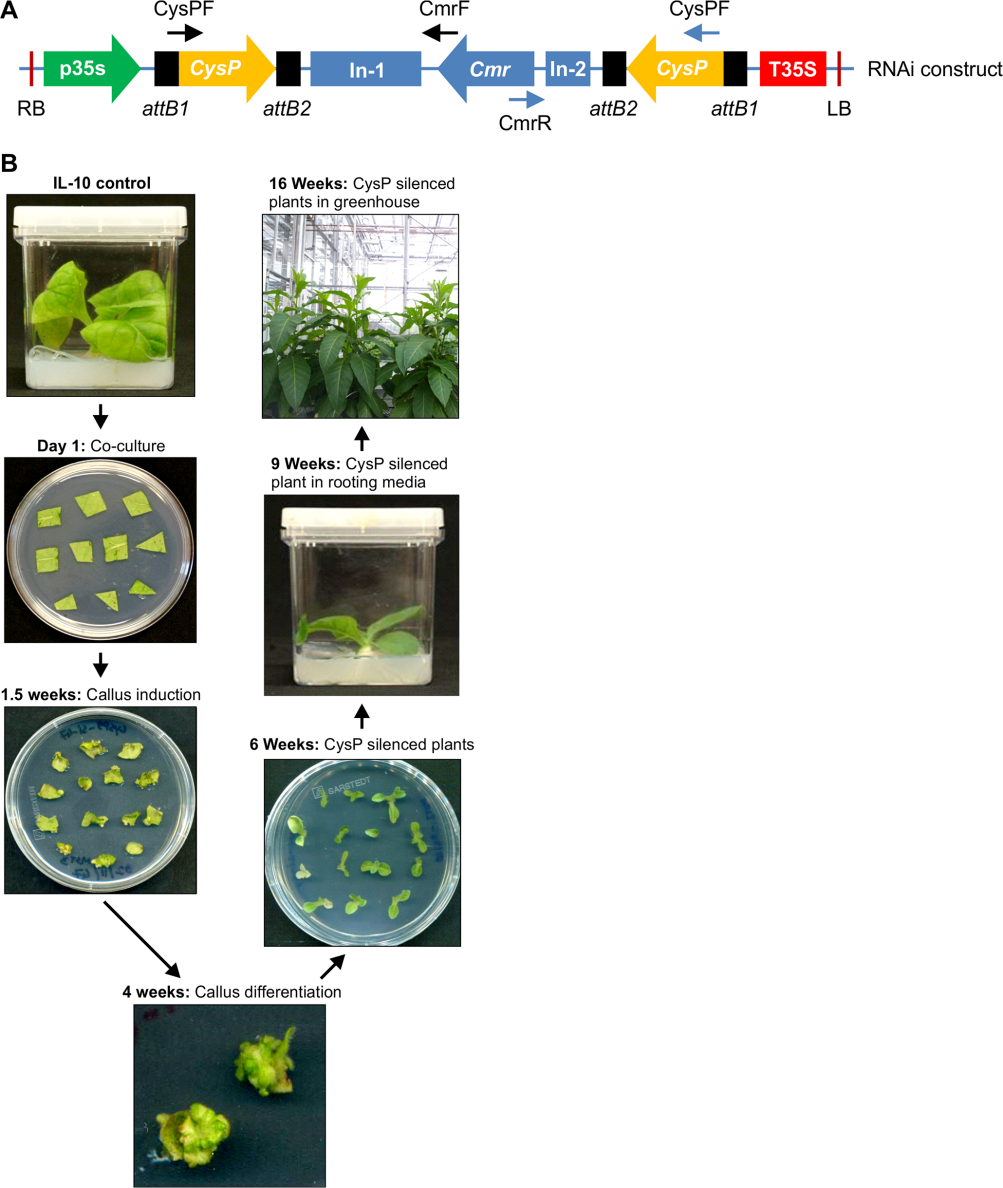
RNAi silencing in tobacco. RNAi constructs were generated for all candidate *CysP*s and the presence of *CysP* double inserts in the silencing vectors were confirmed using primer combinations as shown by the arrowheads (A). Stable *CysP* silenced tobacco lines were generated using the RNAi constructs (B). G7 tobacco lines overexpressing IL-10 protein were grown in magenta boxes. Leaf discs were cut and co-cultured with the *Agrobacterium* cultures containing the RNAi construct. Calli were regenerated from transformed cells and subcultured in differentiation medium containing BASTA. Independent *CysP* silenced transgenic lines were obtained from the individual callus and transferred to the rooting media before transferring them to the greenhouse.

To identify *CysPs* that influence IL-10 accumulation in tobacco, RNAi silencing of selected *CysPs* was performed. Unique regions were identified for each of the *CysP* sequences to facilitate targeted gene silencing. RNAi vectors with the specific *CysP* inserts were screened using two different primer combinations by PCR, which produced amplicons of different sizes. The first amplicon contained the larger intron (In-1) and the second amplicon contained the smaller intron (In-2) region (Fig 3A). A low alkaloid tobacco G7 line constitutively overexpressing IL-10 [32] was used for transforming the silencing constructs. Fig 3B outlines the process of generating *CysP* silenced tobacco plants and the approximate time required to proceed from one stage to the other. In approximately 16 weeks from initial transformation, fully grown plants were obtained. A total of 82 independent transgenic lines were generated, and utilized for the evaluation of *CysP* transcript and IL-10 levels.

In addition to the targeted silencing of specific candidate *CysPs*, a conserved region of *CysP1* to *CysP5* genes was also chosen for silencing. Two silenced lines were generated for *CysPAll-Si*. Since no RNAi line could be generated for *CysP5*, further work on this gene was not carried out.

### Silencing of *CysP6* increases IL-10 accumulation

**Fig 4:**
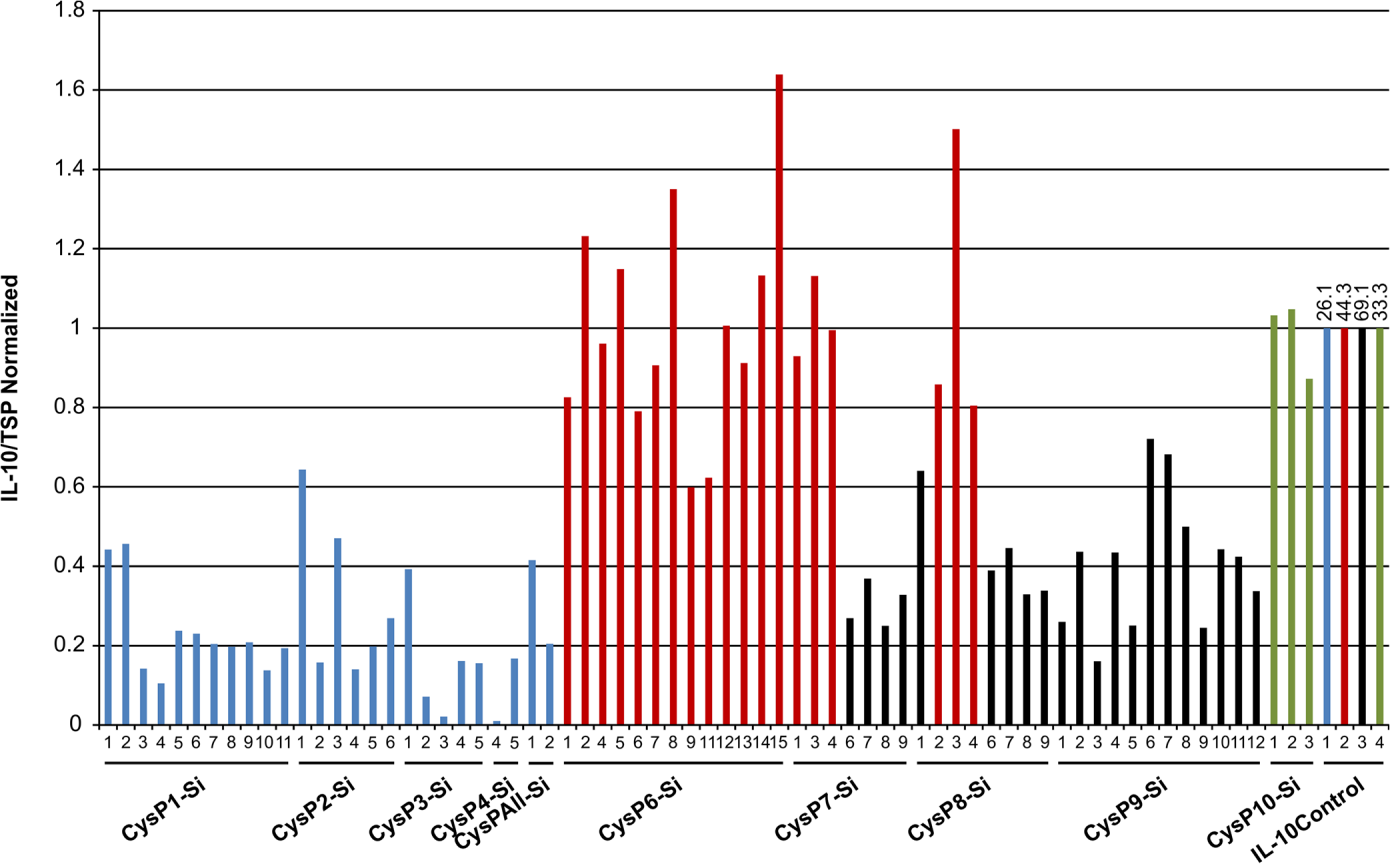
IL-10 accumulation in candidate *CysP* silenced T_0_ tobacco lines. IL-10 accumulation remains lower in comparison to the controls in T_**0**_ generation of *CysP1*-*CysP10* (except *CysP6*) silenced lines. Y-axis represents the normalized value of IL-10/TSP for different independent transgenic lines grown at different time period. Blue, red, black and green bars are normalized against their respective color coded IL-10 level in IL-10 control plant. Numbers above the bar represent actual IL-10 level in ng/mg of total soluble protein in control plants. Si, silenced.

To determine the effect of *CysP* silencing in the accumulation of recombinant IL-10, we measured IL-10 levels in multiple independent transgenic silencing lines for *CysP1-CysP4*, *CysP6-CysP10* and CysPAll. Except for several CysP6Si lines and CysP8Si-3, there was no increase in IL-10 accumulation compared to the IL-10 control plants in any other lines (Fig 4). Since these *CysP* silenced lines were generated over different periods of time, IL-10 control plants were also grown at the same time for an appropriate comparison of IL-10 levels. It was found that the accumulation of IL-10 varied among the controls, even though the plants were grown under similar conditions. Thus, IL-10 accumulations for different T_0_ lines are shown in normalized fold levels.

**Fig 5:**
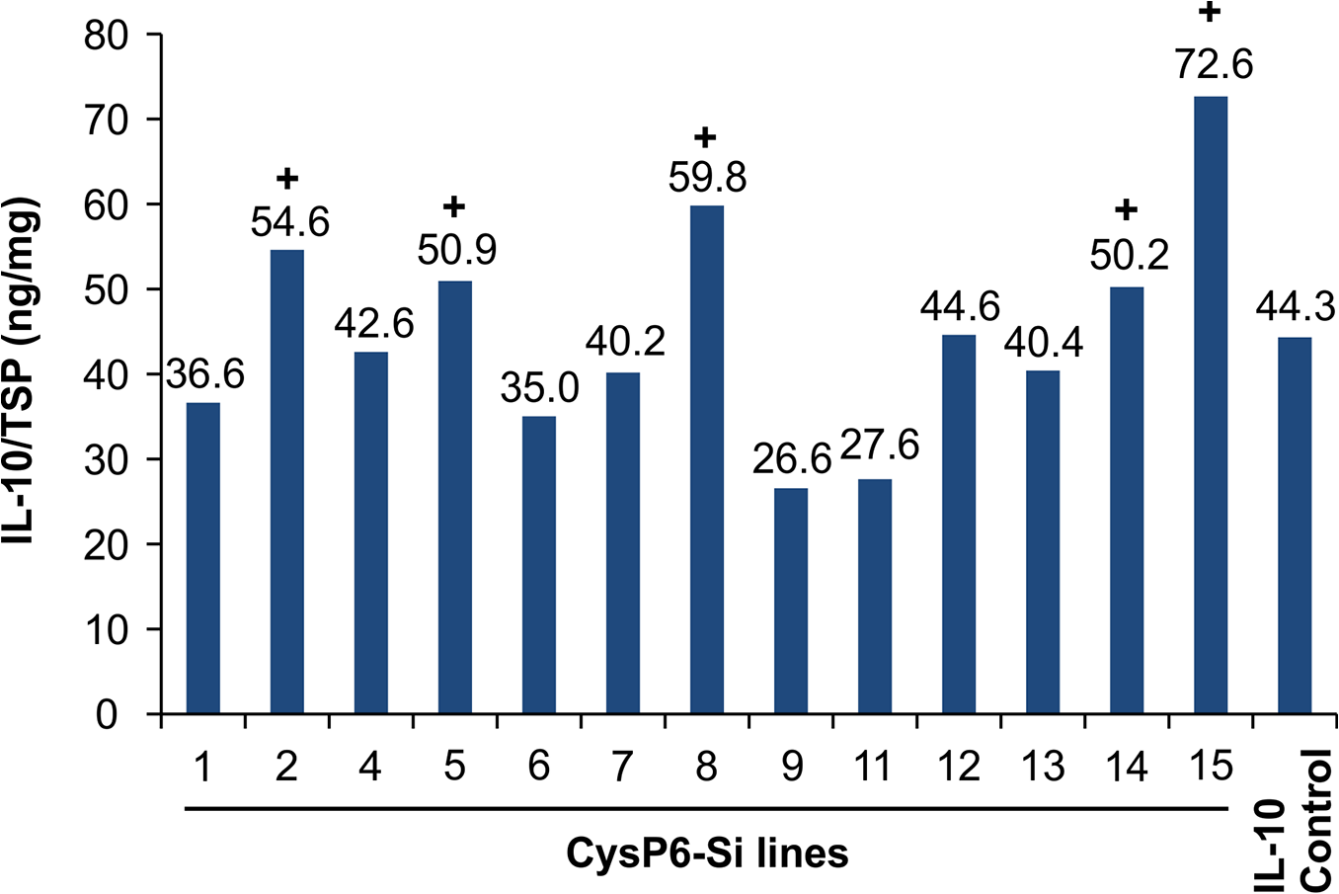
IL-10 accumulation in *CysP6* silenced T_0_ tobacco lines. Measurement of IL-10 level in independent T_**0**_ *CysP6* silenced lines by dsELISA. Data label above the bar represents IL-10 level of individual *CysP6* silenced lines expressed as ng/mg of total soluble protein. **+** indicates the *CysP6* silenced lines with higher level of IL-10 accumulation in comparison to the IL-10 control. TSP, total soluble protein; Si, silenced

A closer look at CysP6Si lines revealed that IL-10 levels were higher in 5 out of 13 *CysP6* silenced lines compared to the control (Fig 5). IL-10 level was increased by 1.6- fold in CysP6Si-15 as compared to the control. Three lines, CysP6Si-4, CysP6Si-7 and CysP6Si-13, displayed similar level of IL-10 accumulation in comparison to the control. The results indicated that silencing of *CysP6* positively affects IL-10 accumulation in T_0_ transgenic tobacco lines.

### Transient silencing of *CysP6* increases IL-10 accumulation

**Fig 6:**
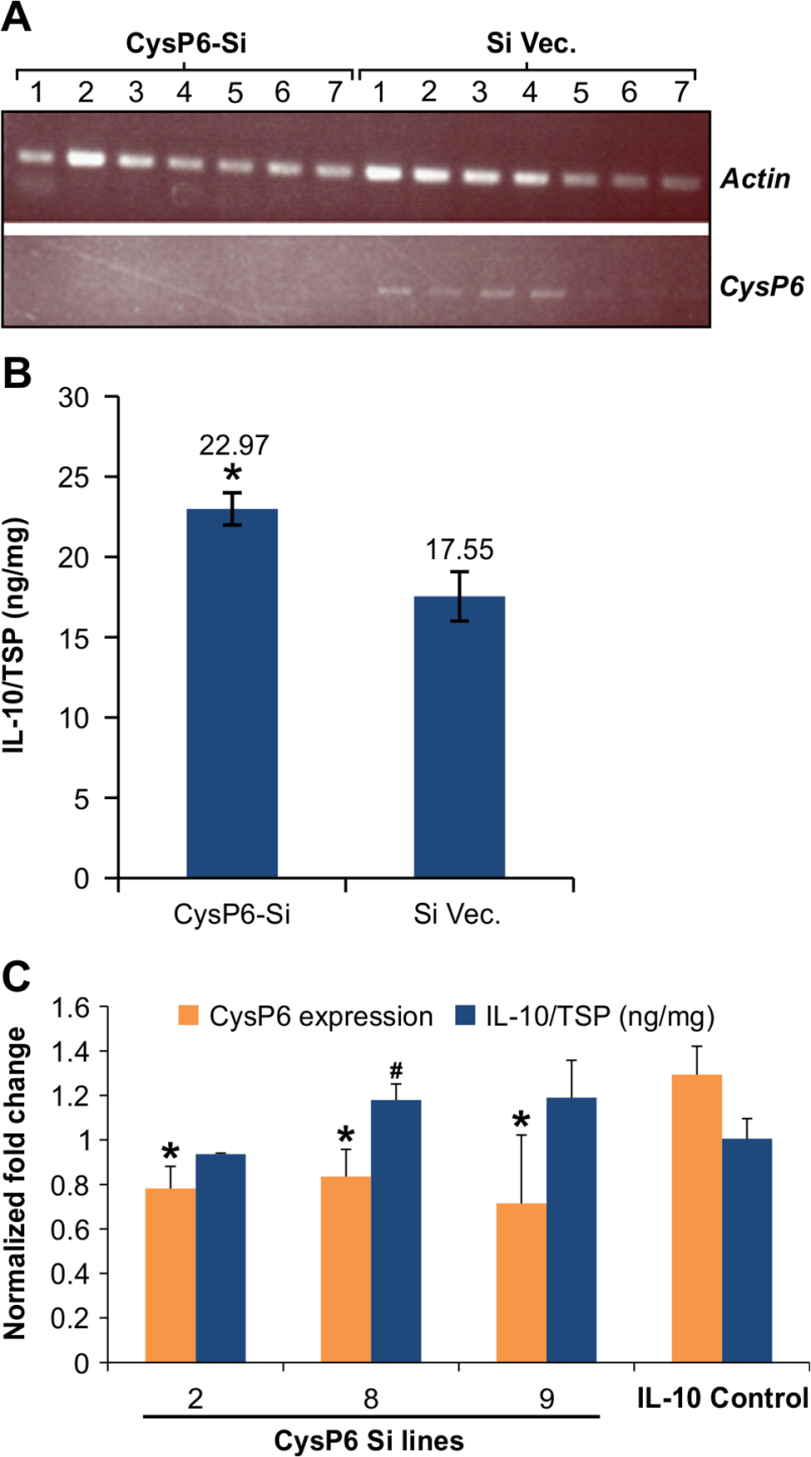
Silencing of *CysP6* in tobacco using transient assay and stable transgenic lines. Leaves from seven weeks old G7 tobacco plants (4^th^, 5^th^ and 6^th^ from the top) were infiltrated with the *CysP6* silencing (CysP6-Si) and empty vector constructs (Si vec.), and the tissues were used to determine the *CysP6* transcript and IL-10 accumulation levels. *CysP6* expressions were checked in CysP6-Si and Si Vec. infiltrated tissues, 4 days post infiltration. The numbers 1–7 indicate the biological replicates (B). The accumulation of IL-10 was measured in all the *CysP6* silencing and vector only infiltrated tissues. The IL-10/TSP values are average of 7 biological replicates and asterisk (*) represents the significant difference in IL-10 level between CysP6-Si and vector only control using student *t*-test at 95% confidence level. Si, Silenced; Vec., Vector (C). Comparison of *CysP6* expression and IL-10 accumulation in CysP6Si T_**1**_ lines. Blue bars represent IL-10/TSP normalized to IL-10 control and orange bars represent fold expression of *CysP6* normalized to *Actin*. Three biological and three technical replicates were used. Error bars represent the standard errors of the biological and technical replicates (D). Asterisks (*) indicates the significant difference in *CysP6* expression as compared to control, and hashtags (#) indicates the significant difference in IL-10 levels between CysP6-Si lines and IL-10 controls using Mann-Whitney *U* test (for qPCR data) and students *t*-test (for ELISA data) at P < 0.05.

To further examine the effect of *CysP6* silencing on IL-10 accumulation levels, transient silencing experiments were performed in tobacco plants that were 1.5 ft tall. *A*. *tumefaciens* containing CysP6-Si construct was infiltrated into tobacco leaf epidermal cells and samples were collected for evaluating *CysP6* transcript and IL-10 protein accumulation. Our RT-PCR analysis using gene specific primers did not detect any *CysP6* transcript in all 7 biological replicates whereas it was present in vector-only plants (Fig 6A). This suggests that *CysP6* was efficiently silenced by transient expression of the silencing construct. Furthermore, IL-10 levels were significantly higher in all 7 biological replicates which were infiltrated with the *CysP6* silencing construct, than tissues infiltrated with the vector-only constructs (Fig 6B). These results demonstrated that transient silencing *CysP6* results in the increase in IL-10 accumulation in G7 tobacco lines.

Transcript abundance was also checked in selected stable *CysP6* silenced T_1_ lines that showed higher IL-10 accumulation in T_0_ generation compared to control lines. As shown in Fig 6C, the lines 2, 8 and 9 showed reduced *CysP6* expression in comparison to the IL-10 controls. *CysP*6 silencing significantly influenced IL-10 accumulation in the lines 8. However, IL-10 level was not altered significantly with the reduction of *CysP6* transcript in the line 2 and 9. Despite this variability, we conclude that IL-10 accumulation was lower in the lines expressing higher *CysP6* in both transient and stable lines.

### CysP6 localizes to the ER

**Fig 7:**
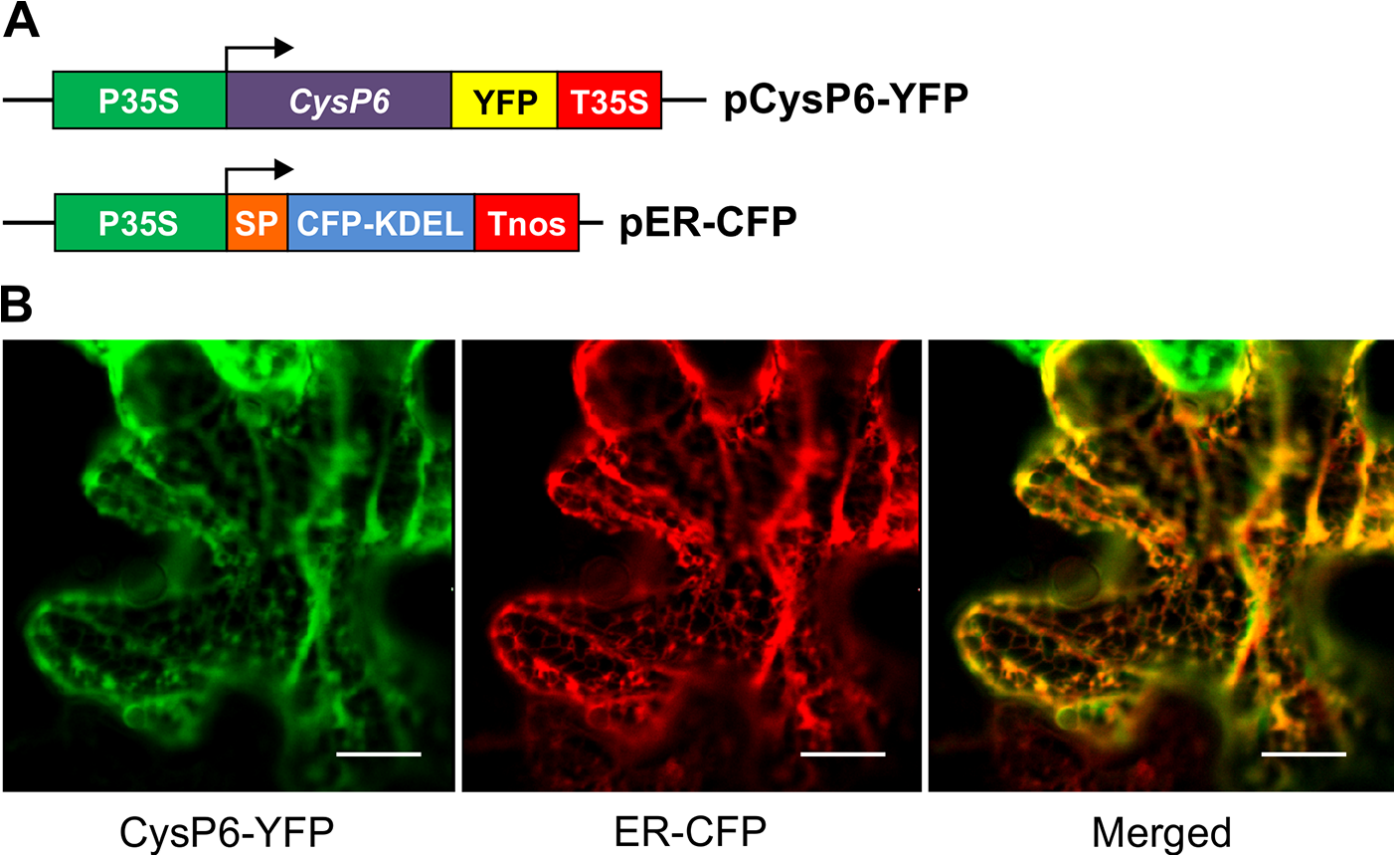
CysP6 localizes to the ER. *Agrobacterium* the carrying plasmids pCysP6-YFP and pER-CFP (ER control) were infiltrated in *Nicotiana benthamiana* (A). The localization was visualized after 48 hours using confocal microscopy (B). CysP6-YFP was visualized in the networks of the ER and co-localizes with the control, ER-CFP. Scale bars, 15 μm.

To evaluate the possibility that IL-10 is degraded by CysP6 in the secretory pathway, the subcellular localization of CysP6 was determined. A translational fusion of CysP6 and YFP was created (Fig 7A), and transiently co-expressed with ER-targeted CFP (control) in *N*. *benthamiana* leaf epidermal cells, followed by confocal microscopy. A typical ER-pattern showing net-like structures and co-localization of CysP6-YFP protein with ER-targeted CFP was observed, indicating that CysP6 localizes in the ER in tobacco leaf cells (Fig 7B).

### IL-10 accumulation increases with plant maturity in tobacco

**Fig 8:**
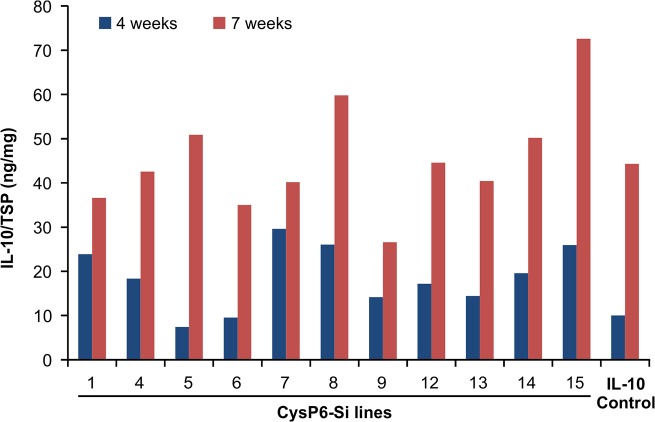
IL-10 accumulation in tobacco plants of different stages. IL-10 accumulation in 4 weeks and 7 weeks old *CysP6* silenced tobacco lines. IL-10 accumulation increased consistently in *CysP6* silenced lines and IL-10 controls with the age. The numbers indicate different independent transgenic lines carrying *CysP6* silencing construct. TSP, total soluble protein; Si, silenced.

To evaluate if the age of transgenic tobacco plants affects the production of recombinant protein, IL-10 levels in *CysP6* silenced lines and IL-10 controls were compared at two different time points. The plants were grown simultaneously in the greenhouse and IL-10 level was measured when they were either 4- or 7-weeks old (time after the transfer of plants from rooting media to soil). The plants were approximately 0.5 ft. tall at 4 weeks of age and approximately 1.5 ft. tall at 7 weeks of age. As shown in Fig 8, the levels of IL-10 accumulation in the leaf tissue increased as plants progressed to maturity in both *CysP6* silenced and IL-10 control plants. Moreover, neither abnormal growth nor any morphological differences were observed for the *CysP* silenced lines compared to IL-10 controls.

### Comparison of IL-10 accumulation in *CysP* silenced T_0_ and T_1_ tobacco lines

**Fig 9:**
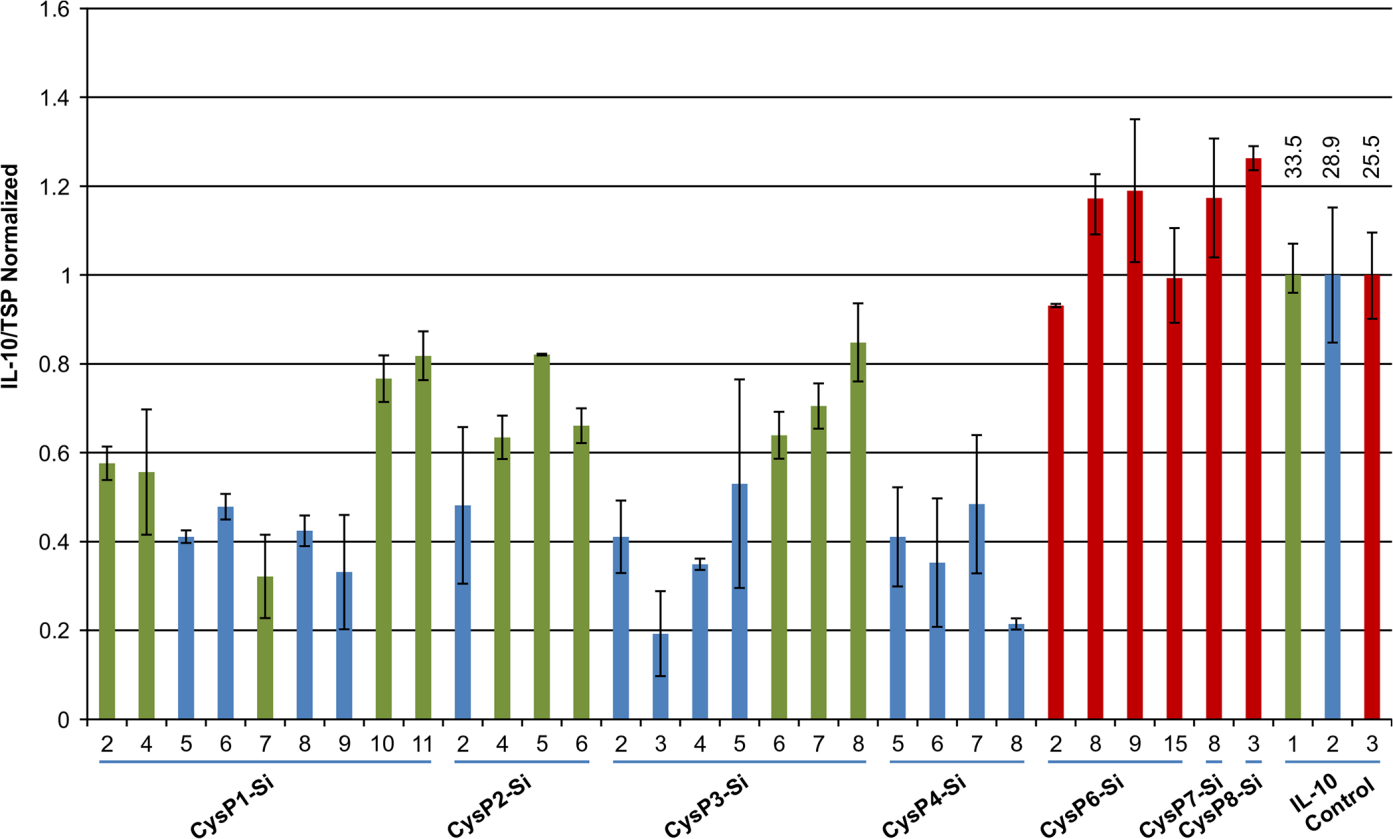
IL-10 accumulation in *CysP* silenced T_1_ tobacco lines. Normalized IL-10 levels of different CysP-Si T_**1**_ lines and controls. Green, blue and red bars are normalized to the IL-10 levels in their respective control plants (IL-10 control, on the right). Numbers above the bar represent the actual IL-10 levels in ng/mg of total soluble proteins. Error bars represent the mean of at least two biological replicates for all the lines except for CysP6-Si lines 8 and 15 (3 biological replicates).

To study the stability of IL-10 accumulation in *CysP* silenced tobacco plants, IL-10 levels were measured for a total of 30 different lines in both T_0_ and T_1_ generations. The accumulation of IL-10 was stable in most of the lines over successive generations of tobacco (Fig 9). *CysP* silenced T_1_ lines, CysP1-Si, CysP2-Si, CysP3-Si and CysP4-Si, all showed lower levels of IL-10 in comparison to their respective IL-10 control plants (represented by same color bars in Fig 9). Lower IL-10 accumulation was also seen for these lines in the T_0_ generation (Fig 4). Similarly, *CysP6* silenced T_1_ lines showed nearly equal (lines 2 and 15) or higher (lines 8 and 9) levels of IL-10 in comparison to the respective control plant. Both CysP6-Si lines 2 and 15 accumulated higher IL-10 in T_0_ generation (Fig 9).This suggests that, the effect of *CysP* silencing on IL-10 accumulation in tobacco remains similar over generations.

## Discussion

Tobacco *CysPs* belong to a large gene family with at least 60 members, and is comparable to that of other plants such as *Arabidopsis* (38 *CysPs*) and *Populus* (44 *CysPs*) [44]. Evolutionarily, tobacco (*N*. *tabacum*) originated from an interspecific cross of two wild forms, *N*. *sylvestris* and *N*. *tomentosiformis*. The interspecific cross between two diploid species with equal number of chromosomes (12 pairs each) resulted into a fertile amphiploid carrying a total of 24 pairs of chromosomes [45]. The larger number of *CysPs* in tobacco could have resulted from the preservation of *CysPs* from both of the parents. CysPs play critical roles in plant growth and development and during responses to different biotic and abiotic factors. Such roles for CysP have been reported in many plants including tobacco [23,38,46–49]. To identify the *CysP* genes that may be involved in IL-10 degradation, expressed tobacco *CysP*s were first identified using the TGI database. Since the tobacco genome sequence has not been published yet, the TGI database was the only major source of information regarding gene sequences and their tissue specific expression. A total of 60 putative *CysP*s were identified from the database and the literature search. The total number of *CysPs* may increase as more unique ESTs are sequenced or as the whole genome sequence of tobacco becomes available.

The papain family tobacco CysPs contained a long propeptide sequence (38–250 amino acids) and a papain domain (220–260 amino acids) (Table 3). Activation of these enzymes can occur by an intra- or inter-molecular proteolysis, where the propeptide sequence is cleaved off [39]. The propeptide sequence also showed a conserved ERFNIN motif which is shared by CysPs from diverse group of species [43]. Mammalian cathepsins with ERFNIN are known to carry out different intracellular and extracellular functions that require the right balance of enzyme activation. Intracellularly, they function in apoptosis and antigen processing, whereas extracellularly, they contribute directly to the degradation of foreign proteins and the extracellular matrix [50]. The signature ERFNIN motif is thought to be associated mostly with inhibiting protease activity before enzyme activation is required. CysP6 and CysP8 contain an extra GRN motif, the role of which in plants is not well understood, but is implicated in protein-protein interactions, targeting and/or activity regulation inside the cell [41].

Quantitative analysis of IL-10 levels in *CysP6*-silenced tobacco lines showed increased accumulation compared to the IL-10 control. Some *CysP6*-silenced lines showed no change in IL-10 accumulation while others accumulated lower levels in comparison to the IL-10 control plants. The accumulation of recombinant protein varied across different *CysP6* silenced lines, both in T_0_ and T_1_ generation. The variation in the IL-10 levels could have resulted from genomic position effects of the inserted *CysP* or differential levels of *CysP6* gene silencing. Such a position effect is a common character of RNAi-mediated silencing and has been described in several transgenic plants and animals [51]. Multiple genes were silenced in *Arabidopsis* using different RNAi constructs, which showed significant transcript variability between the independent RNAi lines of the same target gene [52]. In tobacco, the degree of gene silencing was analyzed in three independent lines carrying *β*-*Glucuronidase* (*GUS*) transgenes, and it was found that the amount of antisense *GUS* RNA correlated with the extent of post-transcriptional silencing in each line [53]. Differential silencing was also observed in the expression of rice *CysP*s, as small interfering (si) RNA was targeted to silence *Rep1* and *EP3A* in transgenic rice cell lines expressing recombinant hGM-CSF [22].

Transient expression of the *CysP6* RNAi construct resulted in near to invisible levels of *CysP6* transcript and significantly increased the accumulation of IL-10 in tobacco. This also supports the fact that *CysP6* is involved in regulating IL-10 accumulation in stable tobacco lines. With the post-genomic era and a flooding of gene sequences, several ways have been explored to assign putative gene functions. Because of its efficiency and specificity, RNAi is one of the widely used techniques in determining gene function. Most of the RNAi approaches utilize generation of stable transgenic lines and observation of RNAi phenotypes related to the particular genes. However, generation of stable lines usually requires a long time, which may range from 2–6 months depending on the type of plants used (4 months in tobacco, Fig 3B). Using *Agrobacterium*-mediated transient expression of the silencing construct, gene functions can be determined in a short period of time. As seen in this study, transient silencing of *CysP6* could be achieved within a week, which is very short compared to the time required to generate stable transgenic tobacco lines. Transient silencing of indigenous genes via agro-infiltration had been reported so far in plant species such as *Vitis vinifera* [54], but not in tobacco. Particularly with the results seen here, transient RNAi can be encouraging for gene functional assays in tobacco.

Silencing of the candidate *CysPs* (*CysP1-CysP5* and *CysP7-CysP10*) in tobacco did not increase IL-10 accumulation. Surprisingly, in all of these *CysP* silenced T_0_ independent transgenic lines, the level of IL-10 accumulation was lower than that of the IL-10 control plant. A detailed transcript analysis of all candidate CysPs may shed light on the extent these *CysPs* are silenced, and how the silencing relates to IL-10 accumulation seen in the individual silenced lines.

CysP6 fused to YFP showed that it is localized to the ER. It was interesting to see the localization pattern of CysP6, without the presence of the C-terminal ER retrieval signal sequence KDEL or HDEL (Fig 2). Several ER resident proteins that lack the retention signal are known to localize in the ER in a signal-independent fashion [55]. *Arabidopsis* RD21 is one of such papain protein without KDEL sequence, and is localized to the vacuole and ER in response to osmotic stress and wounding [55]. Some proteins retained in the ER also play a role in quality control through an interaction with ER chaperones, whose function is to retain and subsequently degrade assembly-defective proteins [56]. Localization in the ER could also suggest similar functions for CysP6 in tobacco.

Though recombinant proteins have been targeted to the ER for a higher yield, their production has been reported to be affected by plant proteases [57]. It was shown that IgG1 targeted to accumulate in the ER is possibly degraded by acidic proteases, most of which consisted of senescence associated CysPs [57]. Localization of CysP6 to the ER provides a good argument for its involvement in IL-10 degradation, since IL-10 accumulates in the ER as well. The degradation process might be occurring along the secretory route when IL-10 is being processed or accumulated. However, a detailed study is required regarding the fate of IL-10, to show that IL-10 degradation occurs within the ER thus affecting its overall yield in tobacco. This study identified and down-regulated several tobacco CysPs, and found CysP6 most likely to be IL-10- specific, is the initial step towards this goal.

## Supporting Information

S1 Table List of primers used for silencing of *CysP*s. (DOCX)
