# Supplementary material for: Identification, Characterization and Down-Regulation of Cysteine Protease Genes in Tobacco for Use in Recombinant Protein Production
Source: PLoS One. 2015 Jul 6;10(7):e0130556. doi: 10.1371/journal.pone.0130556 (PMC4493103; doi:10.1371/journal.pone.0130556)
Supplement: S1 Table — (DOCX) [file pone.0130556.s001.docx]

**Supporting Table 1. List of primers used for silencing of *CysP*s.**

| **Primer name** | **Sequence (5'-3')** | **Primer annealing temperature (**^0^**C)** | **Amplicon size (base pairs)** |
| --- | --- | --- | --- |
| ***Silencing primers*** |  |  |  |
| CysP1F | CCA CCA TAA ACA ATC ATC CAA TCC GT | 52 | 280 |
| CysP1R | TAC CTC TTC CCA TAC CTG TGA GC |  |  |
| CysP2F | TCC GTA CTT TTC TAG GAG CTT CAC | 52 | 242 |
| CysP2R | TGA CTA GTG TCA CAG TCG ACA AG |  |  |
| CysP3F | CAA CTC ATA GCT CCA CTC TCA CCA | 52 | 178 |
| CysP3R | TCC ATG TTC GAG TAG CCA TGA CTC |  |  |
| CysP4F | GGC TAG CTA GTG GTT CAT AAT GGC | 60 | 280 |
| CysP4R | CCA CGC CGA GTT TAT ACC CTT |  |  |
| CysP5F | ATC CCT TCC CCA CAT TCT CTG A | 52 | 249 |
| CysP5R | TAC CTC TTC CCA TAC CTG TGA GC |  |  |
| CysP6F | GCG ATA CTT CCT ACA ATG ACG GTT G | 54 | 345 |
| CysP6R | CAT CTC ACT ACC ATA TCC ACA GCA TCA |  |  |
| CysP7F | CGT GGG ATA CTC TTC AGA TGA CTT GAC | 52 | 204 |
| CysP7R | TCA GCA AAC TCA TTT AGA CCA AGC C |  |  |
| CysP8F | GAA TGG AAA CAG AAG CAG GGG AAA G | 53 | 281 |
| CysP8R | GGA GGA GCA TCA CAA TAA ACT GGA G |  |  |
| CysP 9F | GCA TGT GGC TGA TGC AGG A | 50 | 255 |
| CysP 9R | CGT GTT CTT CTA AGG GCA |  |  |
| CysP10F | GTC CGT AAA ACC CAA AAC CAC AC | 52 | 382 |
| CysP10R | GCT TCT CCA TCT CTC ATA CAA CTC C |  |  |
| ***CysP6-RT-PCR and localization primers*** |  |  |  |
| CysP6-OE-F | GGC **ATG** GCA ACT CTT AGC TTT ACT C | 52 | 1401 (with F/R) |
| CysP6-OE-R | **TCA** AGA ACT GCT CTT CTT TCC TCC ATT |  |  |
| CysP6-OE-R1 | AGA ACT GCT CTT CTT TCC TCC ATT |  | 1398 (with F/R1) |
| **Chloramphenicol resistance (*Cmr) primers*** |  |  |  |
| CmrF | CGA TTC AGG TTC ATC ATG CCG TCT | 55 | variable |
| CmrR | TGA GCA ACT GAC TGA AAT GCC TCC |  |  |

***F, forward primer; R, reverse primer.**

**Supporting Table 2. Comparison of published tobacco *CysP* sequences with tentative contig sequences from tobacco EST database.**

| ***CysP* genes** | **Accession #** | **Closest tentative contig^a^** | **Amino Acid identity (%)** | **Reference** |  |
| --- | --- | --- | --- | --- | --- |
| *NtCP1* | | AY881011 | **TC133645 (*CysP4*)** | 94.84 | ([Beyene et al., 2006](#_ENREF_11)) |
| *NtCP2* | | AY881010.1 | **TC124565 (*CysP2*)** | 96.6 | ([Beyene et al., 2006](#_ENREF_11)) |
| *CyP7* | | Z13959.1 | TC131927 | 97.8 | ([Linthorst et al., 1993](#_ENREF_61)) |
| *CyP8* | | Z13964.1 | TC131927 | 97.5 | ([Linthorst et al., 1993](#_ENREF_61)) |
| *NtCP56* | | EU429306.1 | **TC166795 (*CysP10*)** | 99.17 | ([Zhang et al., 2009](#_ENREF_107)) |

^a^ Closest tentative contigs in bold letters (candidate CysPs in parenthesis) share a high degree of amino acid identity with the respective published CysP sequences.
